# Exploring the Potential of Metatranscriptomics to Describe Microbial Communities and Their Effects in Molluscs

**DOI:** 10.3390/ijms232416029

**Published:** 2022-12-16

**Authors:** Magalí Rey-Campos, Raquel Ríos-Castro, Cristian Gallardo-Escárate, Beatriz Novoa, Antonio Figueras

**Affiliations:** 1 Institute of Marine Research (IIM), National Research Council (CSIC), Eduardo Cabello 6, 36208 Vigo, Spain; 2 Interdisciplinary Center for Aquaculture Research (INCAR), University of Concepción, Concepción P.O. Box 160-C, Chile

**Keywords:** *Mytilus*, molluscs, microbiome, diversity, metatranscriptomics, RNA-seq, genomics

## Abstract

Metatranscriptomics has emerged as a very useful technology for the study of microbiomes from RNA-seq reads. This method provides additional information compared to the sequencing of ribosomal genes because the gene expression can also be analysed. In this work, we used the metatranscriptomic approach to study the whole microbiome of mussels, including bacteria, viruses, fungi, and protozoans, by mapping the RNA-seq reads to custom assembly databases (including the genomes of microorganisms publicly available). This strategy allowed us not only to describe the diversity of microorganisms but also to relate the host transcriptome and microbiome, finding the genes more affected by the pathogen load. Although some bacteria abundant in the metatranscriptomic analysis were undetectable by 16S rRNA sequencing, a common core of the taxa was detected by both methodologies (62% of the metatranscriptomic detections were also identified by 16S rRNA sequencing, the Oceanospirillales, Flavobacteriales and Vibrionales orders being the most relevant). However, the differences in the microbiome composition were observed among different tissues of *Mytilus galloprovincialis*, with the fungal kingdom being especially diverse, or among molluscan species. These results confirm the potential of a meta-analysis of transcriptome data to obtain new information on the molluscs’ microbiome.

## 1. Introduction

Although it might seem otherwise, the marine environment can be a source of potential pathogens for the inhabiting organisms. For instance, it is estimated that 10^23^ viral infections occur every second in the oceans [1]. Similarly, high concentrations of bacteria are also present in marine environments, with an estimated 10^6^ cells per mL [2]. Studies aiming to understand the interactions between these microorganisms and marine metazoans have been performed [3,4], especially in commercial species [5,6] and species with ecological value, such as sponges, in which microbiome studies have allowed to assay the impact of pollution [7,8]. However, in marine environments, in addition to bacteria and viruses, eukaryotes, such as protozoans and fungi, are also in close contact with marine metazoans and can interfere with their natural development.

Although some microorganisms may be pathogenic, they can also be beneficial, which is why they are present in different parts or tissues of multicellular beings and comprise their microbiomes [9,10,11]. Therefore, the study of the microbiomes of marine animals has gained importance from a biological point of view, given the particularity of the environment that surrounds them. It is also important to highlight other interesting factors that motivate these studies, such as the filter-feeding biology of bivalves, which are especially exposed to potential pathogens living in the water column, or the high immune resistance of mussels, of which no mass mortalities have been reported in the field [12]. This scenario raises the question of the impact that the basal microbiome of a marine filter-feeding animal exerts on its immune status and its ability to react to new pathogenic stimuli.

The microbiomes of different animals have been widely explored with well-established technologies, such as 16S and 18S rRNA gene sequencing, which allows the identification, classification, and quantification of the prokaryotic and eukaryotic communities of the considered samples [13,14]. In relation to the bivalve microbiome, the bacterial profiling of the gut and haemolymph after micro- and nanoplastic exposures [15,16,17], comparing several bivalve species [5] or different temperatures [18] are the most relevant works performed so far in the area

However, there are some methodological difficulties that have not been solved thus far, for example, how to identify viruses. These entities lack conserved ribosomal genes to be amplified using the aforementioned technology, and even so, they require more extensive work due to the great variability and the scarce taxonomic information available. Another issue is the bias of the ribosomal amplicon sequencing that allows the identification of a specific gene in the prokaryote/eukaryote genome, with the benefit of an increased sequencing depth but the limitation of losing more information that could be valuable. For instance, which genes could be expressing the detected microorganisms beyond ribosomal genes.

In this work, we used a metatranscriptomic approach consisting of mapping the RNA-seq reads to all the microorganism genomes of interest to identify, classify, and quantify viruses, bacteria, protozoans, and fungi without the need to produce amplicons in mussels. This strategy also allows a comparison of the mussel microbiome–transcriptome to find some correlation among the gene expression and microorganisms abundances. Finally, a microbiome profile of different mussel tissues as well as haemocytes of different molluscs was performed.

## 2. Results

### 2.1. Comparison between 16S rRNA Sequencing and RNA-Seq Taxonomic Profiling

**Figure 1:**
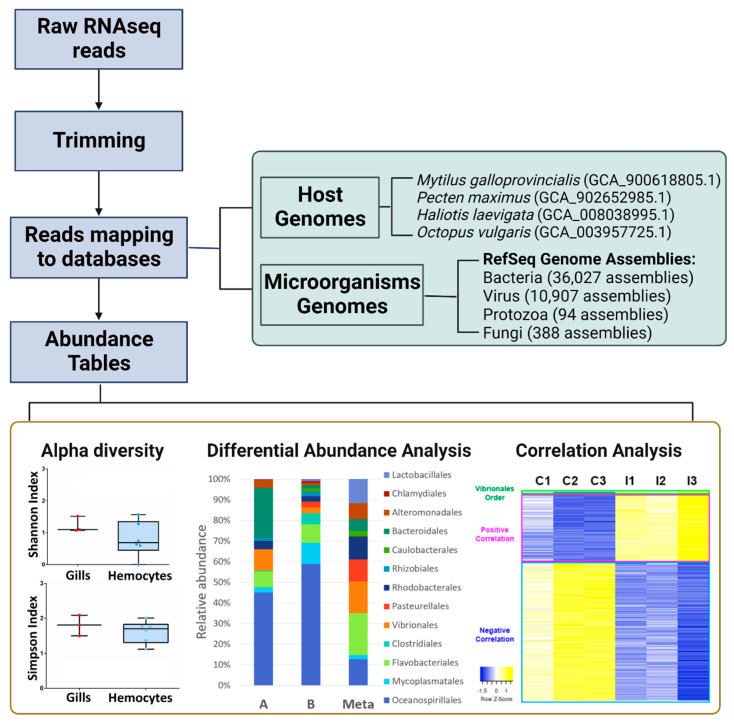
Working plan implemented for the study of metatranscriptomics.

Initially, we questioned whether metatranscriptomics (the working scheme is available in Figure 1) could be a suitable approach in relation to 16S rRNA sequencing; therefore, we compared the two methodologies. Different samples from Ría de Vigo were analysed in parallel and independently.

**Figure 2:**
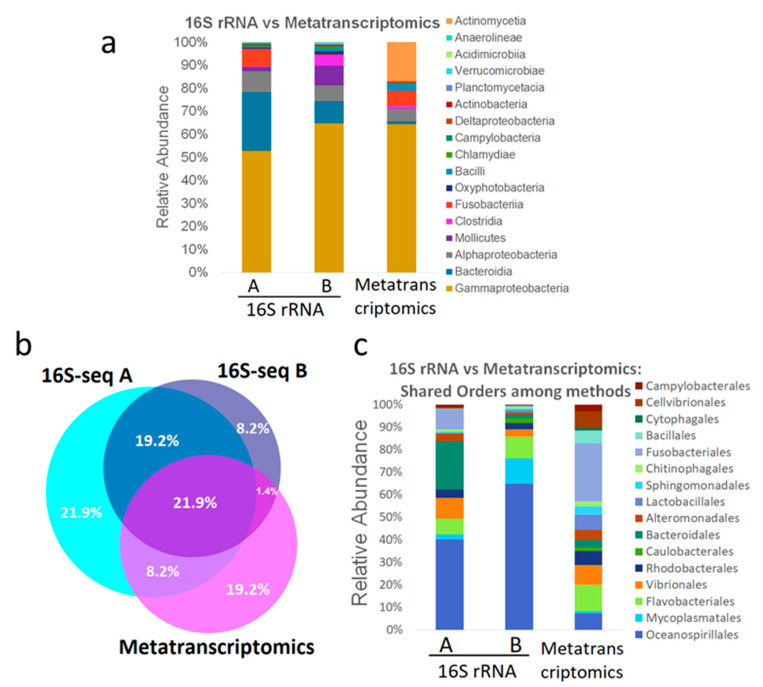
Methodological comparison: 16S rRNA sequencing vs. RNA sequencing. (**a**) Histogram showing the classes detected by 16S rRNA and metatranscriptomics. Taxa with combined abundances over 500 are shown. (**b**) Venn diagram showing the shared and exclusive orders among the three approaches. (**c**) Histogram showing relative abundances of orders shared among the three approaches.

The taxonomic profiling data of the mussel microbiome based on the 16S rRNA sequencing results were compared with the metatranscriptomic data from mussels of the same origin, Ría de Vigo (NW Spain), and analysed to determine the bacterial diversity (the data details are in Supplementary Table S1 and the sequencing general statistics can be accessed in Supplementary Table S2). Although the samples were derived from different individuals, several similarities were observed between the samples sequenced by 16S rRNA and with the results obtained through the metatranscriptome sequencing (Figure 2a). The most abundant classes (Gammaproteobacteria, Bacteroidia, Alphaproteobacteria, Mollicutes, Clostridia, Fusobacteriia, and Bacilli) were detected by both methods. However, other classes, such as Actinomycetia, Betaproteobacteria, and Flavobacteriia, were especially abundant in the metatranscriptomic study and undetectable in the 16S rRNA sequencing results (Figure 2a).

The similarities aforementioned were also revealed at the order level because a core of shared taxa among the three sequencing experiments was observed (Figure 2b). This core of shared orders is shown in Figure 2c, in which the relative abundance of the 16 orders detected by three approaches is represented. These orders include Oceanopirillales, Mycoplasmatales, Flavobacteriales, and Vibrionales.

This methodological comparison was also performed by working with the information available in public databases. The work of Iori et al., 2020 [19], included RNA-seq and 16S rRNA sequencing data from the same samples. Upon reanalysing these data, it was possible to make a comparison between the two approaches, concluding the same as the above described (Supplementary Figure S1).

Once we confirmed the usefulness of the metatranscriptomic pipeline to describe the bacterial diversity in mussel samples, we used it to reanalyse the mussel transcriptomes from our group and from public BioProjects that included 24 SRA sets of data/reads (comparing tissues, stimuli, and mollusc species). All the information is available in Table 1 and Supplementary Tables S1–S5.

### 2.2. Microbiome Comparison between Gills and Haemocytes of Mytilus galloprovincialis

**Figure 3:**
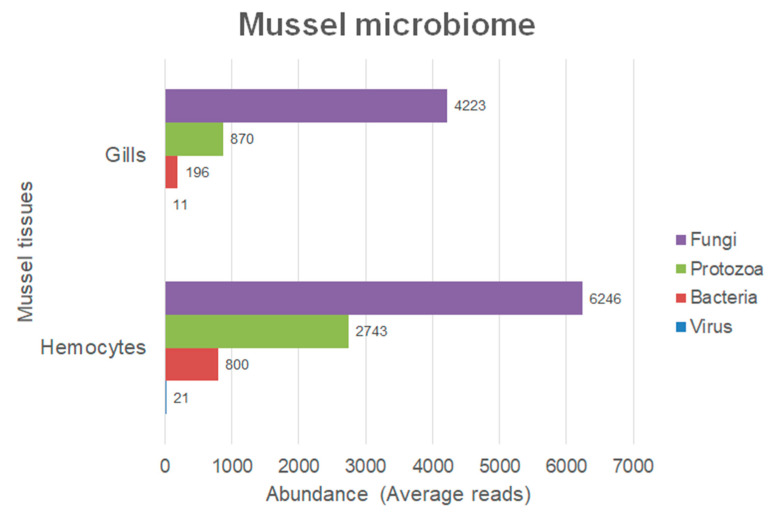
Microbiome comparison of the gills/haemocytes of *M. galloprovincialis*. Numbers indicate the quantity of reads that mapped in each group (on average, 3 individuals in gills and 6 in haemocytes).

First, and because of the importance of these two tissues from an immunological point of view, a comparison of the microbiome between mussel gills (*n* = 3) and haemocytes (*n* = 6) was performed (details in Supplementary Table S3). Bacterial, viral, protozoan, and fungal abundances were analysed in both tissues of *Mytilus galloprovincialis* individuals. The gills and haemocytes showed a similar microbial profile, with members of the fungi group being the most abundant, followed by protozoa, while reads from the viruses and bacteria were notably less abundant (Figure 3).

**Figure 4:**
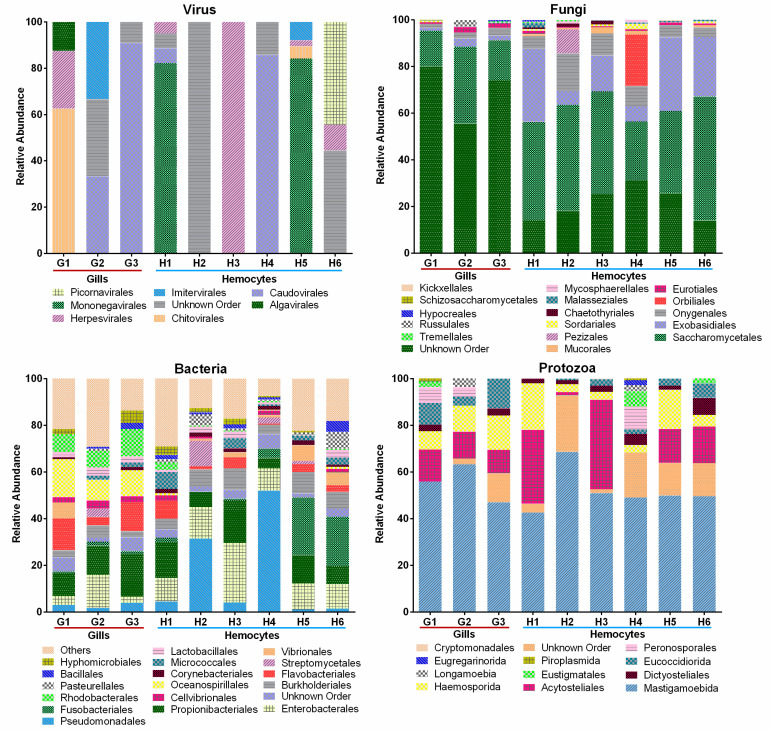
Taxa relative abundances in the gills and haemocytes of *M. galloprovincialis*. The taxonomic category shown is order.

In general, the profiles of the bacteria and viruses seemed more heterogeneous than those of the protozoa and fungi (Figure 4) when considering biological replicates and different tissues. The most abundant bacterial orders were Pseudomonadales, Enterobacterales, Propionibacteriales, and Flavobacteriales. With respect to protozoa, Mastigamoebida prevailed, reaching 50% abundance. There were also other highly abundant orders, such as Acytosteliales and Haemosporidia. The most abundant fungal orders were Saccharomycetales and Exobasidiales.

**Figure 5:**
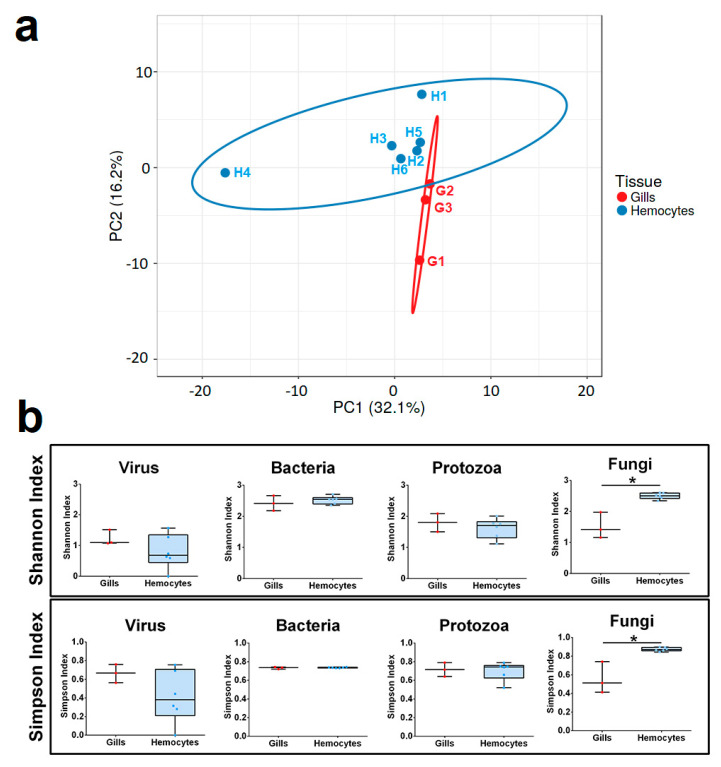
(**a**) PCA describing the dataset containing taxa (all the analysed groups) abundance in the gills and haemocytes of *M. galloprovincialis*. (**b**) Shannon entropy and Simpson’s indexes of viruses, bacteria, protozoa, and fungi in the gills and haemocytes of *M. galloprovincialis*. * indicates a *p* value < 0.05 after the Mann–Whitney U test.

The principal component analysis showed a relative tissue specificity (Figure 5a). Fungi seemed to be the weightiest group according to the Shannon and Simpson indexes of diversity (Figure 5b). None of the other analysed microbial taxa showed differences in terms of the diversity between the haemocytes and gills; therefore, it is worth noting that a core of microorganisms was found shared between all nine studied transcriptomes (Supplementary Figure S2). In particular, the most abundant taxa across all the samples were the Saccharomycetales fungal order, followed by the Mastigamoebida protozoan order (Figure 4).

### 2.3. Effect of a Pathogenic Stimulus on the Microbiome Composition of M. galloprovincialis and Gene Expression

After studying the gill microbiome as the first defence barrier and the haemocytes as the central immune cells in mussels, we wanted to study the effect of a bacterial infection in the microbiome of *Mytilus galloprovincialis*. The transcriptomes associated with two types of infection with *V. splendidus* were taken into account: the gill transcriptome after a bath infection [20] and the haemocyte transcriptome after an intramuscular injection [21] (details of the samples in Supplementary Table S4).

None of the infections produced any microbiome changes in either the tissue, gills, or haemocytes. Therefore, the full microbiome composition did not change after *V. splendidus* infection. As expected, the reads of *Vibrio* spp. were significantly increased in the infected samples compared with those in the controls.

**Figure 6:**
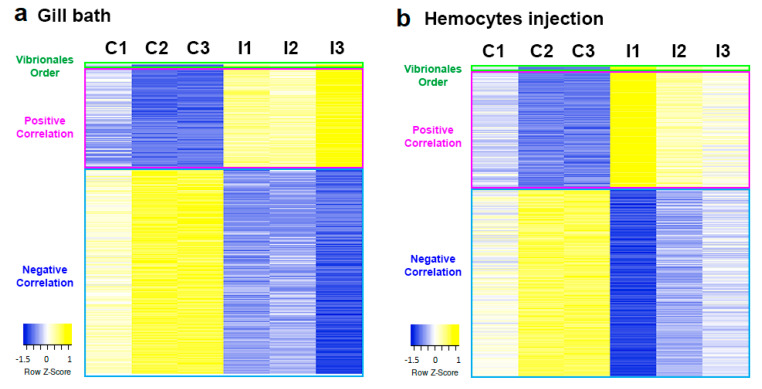
Correlation between Vibrionales abundance and mussel transcriptome data. Heatmaps show Vibrionales order abundance values and TPM values of the highest correlated transcripts (R^2^ > 0.90). A logarithmic transformation of the data was performed ((log2(x + 1))). Graph (**a**) corresponds to the bath infection experiment [20] and graph (**b**) to the injection experiment [21].

We aimed to determine which transcripts correlated with the abundance of this targeted taxa. By performing a Pearson correlation analysis of the abundances of the Vibrionales order in each sample and the expression values obtained by the RNA-seq analysis, we obtained a strongly correlated group of genes. Figure 6 shows the results of both experiments with the Vibrionales abundance as the first line of the heatmap, followed by all the transcripts with an R^2^ higher than 0.9.

Among the top 20 annotated and correlated genes (Table 2), it can be observed that the higher bacterial loads induced a more inhibited gene expression. Moreover, even though the genes modulated in each of the experiments were different, there were notable similarities in the functions that they perform. The main processes that negatively correlated with the bacterial load are involved in molecular processes such as the initiation of transcription (zinc finger proteins, C-Myc-binding protein, or TATA box-binding protein-associated factor RNA polymerase I subunit C). In the same way, the trafficking processes and therefore the immune routes such as apoptosis and autophagy were also negatively correlated with the abundance of *Vibrio* spp. (regulator of MON1-CCZ1 complex, Ankyrin-1, Sialoadhesin, nuclear pore complex protein Nup54, C-Myc-binding protein, and programmed cell death protein 2).

This approach facilitates the discovery of the processes modulated more closely with the growth of the pathogen. In fact, a comparison of the GO terms enriched in the genes whose expression correlated with the bacterial load and those revealed by a usual analysis of the RNA-seq (DEGs after differential expression analysis) in both experiments showed a strong similarity between the shared processes (Supplementary Figure S3), although the genes were different (Supplementary Figure S4). This result means that this strategy, which is based on correlation analyses with the pathogen growth level, can be complementary, offering a set of genes whose expression changes tightly in relation to the abundance of the pathogen, while a normal RNA-seq analysis would provide a more extensive and global comparison.

### 2.4. Haemocyte Microbiome of Molluscs

**Figure 7:**
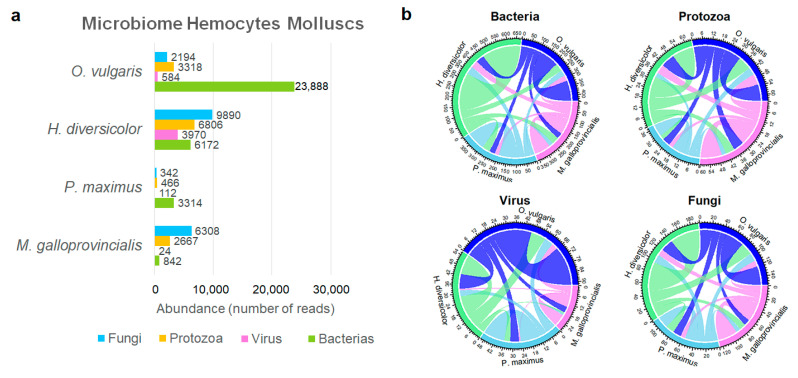
Metatranscriptome comparison between the haemocytes of 4 mollusc species. (**a**) Abundances of each group in the 4 studied species. Numbers indicate the quantity of reads that mapped in each group. (**b**) Chord diagram showing the number of taxa shared by each pair of species.

Finally, to determine the extent of the microbiome specificity of the haemocytes, different molluscs (*M. galloprovincialis*, *Pecten maximus*, *Haliotis laevigata*, and *Octopus vulgaris*) were compared. Supplementary Table S5 provides the information about these sample reads. Figure 7a displays the abundance of each metatranscriptome group in each studied mollusc species. *Octopus vulgaris* showed a significantly higher abundance of bacteria than the others. *Pecten maximus* showed the lowest abundance values for both fungi and protozoa. Moreover, the highest viral abundances were found in abalone (10-fold more than the others). Each species showed its own metatranscriptomic profile, which was characterised by an exclusive set of microorganisms, and only a small group was shared with each of the other analysed molluscs (Figure 7b).

## 3. Discussion

16S sequencing has been the preferred technology for the study of the bacterial profiles of marine organisms, particularly bivalves. Although there is not much information about this subject, bacterial diversity has been studied to determine the influence of temperature [18,25] or exposure to pollutants [19] and plastics [15,16,17], with the gut (because of its strict relation to microbiota) and haemolymph (because of the impact on the immune response of these animals) being the most studied tissues. A recent study also reported the possibility of acquiring a microbiome through vertical and horizontal transmission in Sydney rock oysters [26]. However, in recent years, the use of metatranscriptomics to describe microbial variability has gained considerable interest. Shotgun approaches may work as a good and unbiased method to identify microbiota profiles. This idea, proposed in our work, has also been supported in some comparisons in other species in which the authors conclude that RNA-seq and 16S-MiSeq are equally sensitive in detecting bacteria [27] or even that when a sufficient number of reads is available, shotgun sequencing is better able to identify less abundant taxa than 16S sequencing [28]. In fact, we identified several taxa by RNA-seq that were not identified by 16S amplicon sequencing (19.2%, including Actinomycetia, Betaproteobacteria, and Flavobacteriia). Moreover, the other way around, 49.3% of all the detected taxa were only identified by 16S rRNA sequencing. Even so, in terms of evolution, the 16S ribosomal gene offers the potential for reconstructing phylogenies to understand the evolutionary mechanisms of the relation host–microorganisms. Metatranscriptomics show some limitations as the full metatranscriptome assembly can be very challenging if the depth is insufficient; also, some taxa might be transcriptionally inactive being under the threshold of detection.

Our results on the mussel microbiome agree with those reported in a study of bacterial diversity in five different tissues of *M. galloprovincialis* conducted by 16S rRNA sequencing [29]. In this case, it could be observed that each organ showed a high level of specificity and that the haemolymph would have an alpha diversity Simpson index significantly higher than the gills. In our experimental approach (taking into account the different experimental approaches and differences in sample type: DNA vs. RNA), organ specificity, despite being an open circulatory system and a filter-feeding animal, was evident in some of the dominant orders in each tissue (Oceanospirillales in the case of the gills or Flavobacteriales in the case of the haemocytes/haemolymph). However, there is a common base constituted by a series of bacteria that are present in most of the tissues, such as Enterobacterales, Pseudomonadales, or Propionibacteriales.

Although, as mentioned, most of the work in this area has been focused on bacterial identification, because they are filtering animals, many other microorganisms may be present on their tissues, such as protozoans or fungi, as well as viral particles that may have a significant influence on their immune competence. For instance, it is well established that protozoans of the *Marteilia* genus are parasites of bivalves [30,31] and may cause mortalities in the case of unfavourable environmental conditions. However, many others may appear naturally in their microbiome, as may be the case for Mastigamoebida, Acytosteliales, or Haemosporida, which were remarkably detected in the current work both in the haemocytes and gills. With respect to viruses, none have been identified as causing pathological impacts or mortalities in mussels, something that does occur with other closer bivalves such as the Japanese oyster, severely affected by the oyster herpesvirus [32,33,34] and considered a major problem in aquaculture [35,36,37]. To date, several attempts to identify viruses in mussel samples have been made; such attempts have aimed to detect transcripts by identifying viral domains such as RdRp [3,6] or by building viral databases and blasting sequences [6,38]. Similar to this last idea, we present in this work the putative viral identification by mapping reads to all the sequenced viral genomes available in the public databases. The number of reads similar to the virus found using this methodology in mussel tissues was practically negligible; this approach only works for known viral genes, and it is necessary to combine several strategies for proper identification [39]. Nevertheless, it leaves the goal of future work to find an explanation for the viral scarcity in mussel tissues given its filtering biology and the environment that surrounds it, which contains plenty of viral particles [1].

Apart from tissue specificity, the microbiome of a marine organism could be affected by the geographic location or by different environmental conditions. It was reported that *Bathymodiolus azoricus*, sampled in relatively close proximity in the Mid-Atlantic Ridge, showed a high conservation of the bacterial community structure [40]. However, the microbiome differences may be notably influenced by obvious variations in environmental conditions such as temperature, already defined as a decisive factor [18,25,41].

Moreover, when we compared the microbial composition of haemocytes from four different mollusc species (*Mytilus galloprovincialis*, *Pecten maximus*, *Haliotis diversicolor*, and *Octopus vulgaris*), we observed completely different microbial profiles, even though three of the species were sampled in the same geographic area. Haemolymph is one of the most frequent samples considered for the study of microbiome-related subjects because of the aforementioned relevance of the immune cells it contains. External factors, such as exposure to micro- and nanoplastics or changes in temperature, modify microbiota compositions and are accompanied by changes in immune parameters in which haemocytes are involved, such as phagocytosis or ROS/NO production [15,16,25]. Something similar was reported about the bacteria with antimicrobial activities present in several bivalves and echinoderms showing notable differences in both abundance and diversity [4]. There are taxa present in all the studied species, for instance, the *Vibrio* genus. Vezzulli et al., 2018 [5], via a 16S rRNA comparison between *M. galloprovincialis* and *Crassostrea gigas*, reported that *Vibrio* and *Pseudoalteromonas* were the most relevant genera, dominating the whole bacterial profile [5].

In addition to the basal microbiome pattern, external factors (temperature, contaminants, or infections) may also modify the abundance of certain taxa, resulting in, for instance, an increased vulnerability to the entry of opportunistic pathogens [25,41] or general changes in immune processes [15,16]. In our work, the stimulus used for the analysis of gills (in a waterborne infection) and haemocytes (muscle injection) was *Vibrio splendidus* infection. Twenty-four hours after the infection, no modification of the microbiome was found. Something slightly different occurred in the haemolymph of *M. coruscus* [25], in which the *Vibrio cyclitrophicus* infection promoted the proliferation of opportunistic pathogens (Arcobacter and Francisella) but after 8 days of infection and at a temperature of 21 °C (6 °C higher than in our case).

Another important advantage of metatranscriptomics is the identification of genes that modulate their expression in a similar way to the infection process. Then, it is possible to determine a correlation between the host gene expression values and the abundance values of the considered pathogen. This is interesting because it has been described that specific microbes play important roles in regulating the expression of individual host genes [42,43], leading to downstream effects on many biological processes, including host immunity. The main processes that we found to be negatively correlated with the *Vibrio* spp. load are involved in the initiation of transcription and trafficking processes (apoptosis and autophagy). We already discussed in Rey-Campos et al., 2019 [21], and Saco et al., 2020 [20], which processes and genes were modulated after *Vibrio splendidus* infection, but the present analysis showed some new genes (the regulator of MON1-CCZ1 complex, Ankyrin-1, Sialoadhesin, nuclear pore complex protein Nup54, C-Myc-binding protein, and programmed cell death protein 2) that supplement the previous findings. This approach will produce a list of more tightly modulated genes related to the infection levels than those obtained in a general RNA-seq analysis.

Finally, the current study exploits the potential of a meta-analysis of transcriptome data by reusing publicly available information for *M. galloprovincialis* and related mollusc species. A meta-analysis of public data is an essential component of open science and has been facilitated by the ‘big data’ revolution [44]. However, this type of study continues to be difficult due to the large amount of missing data (it was estimated that 56% of ecological and evolutionary databases are incomplete [45]).

In summary, in the current work, we performed a metatranscriptomic study of mussels to obtain a description of the host microbiome and transcriptome. We were able to describe the different bacteria, protozoans, fungi, and viruses that are found in their gills and haemocytes. The practical absence of viral detection is noteworthy. Other microbiome groups (protozoa, fungi, and bacteria) showed differences between the compared tissues. The abundance of a particular microorganism may be well established and correlated with the gene expression of the host, allowing a very strict analysis in relation to, for instance, an experimental infection.

## 4. Materials and Methods

### 4.1. 16S rRNA Sequencing

Before analysis of the microbial diversity obtained with the metatranscriptomic approach, the prokaryotic diversity was evaluated in mussels from Ría de Vigo (NW Spain) by the most frequently used method, that is, sequencing 16S rRNA amplicons. Two mussel samples from Ría de Vigo were analysed independently (different primers for amplification and different sequencing platforms). A total of 6 samples (fragment of the whole body from 15 individuals pooled into a single sample) were taken for the first analysis (16S-seq A), and 9 samples (fragment of the whole body from 3 individuals pooled) were taken for the second analysis (16S-seq B).

DNA from the samples was isolated using the Maxwell RSC Blood DNA Kit (Promega, Madison, WI, USA) according to the manufacturer’s instructions. The DNA concentration was estimated using a NanoDrop™ 1000 spectrophotometer (NanoDrop Technologies, Inc., Wilmington, DE, USA). In the case of 16S-seq A, the V4 region of the 16S rRNA gene (287 bp long) was amplified from the DNA samples using universal prokaryotic-specific primers for the 16S rRNA gene described by Lasa et al. (2019) [46]. A first target-enrichment PCR assay was performed with the 16S conserved primers, followed by a second PCR assay with customised primers, including adapter complementary regions [46]. The 16S-seq A libraries were sequenced using an Ion Torrent (PGM) Platform (Thermo Fisher Scientific, Waltham, MA, USA).

In the case of 16S-seq B, the V3-V4 region of the 16S rRNA gene (~460 bp long) was amplified using universal prokaryotic-specific primers described by Klindworth et al., 2013 [47]. After amplicon purification with AMPure XP beads, a library for 16S metagenomics sequencing was prepared using the Herculase II Fusion DNA Polymerase Nextera XT Index Kit V2 (Illumina), and paired-end sequencing (2 × 300) was performed on an Illumina MiSeq platform (Macrogen, Seoul, Korea).

The obtained raw read sequences were deposited in the Sequence Read Archive (SRA) (http://www.ncbi.nlm.nih.gov/sra, accessed on 14 December 2022) under BioProject accession numbers PRJNA858248 (16S-seq A) and PRJNA858246 (16S-seq B).

The bioinformatic pipeline was performed using the Microbial Genomics module 1.3 of the CLC Genomics workbench 9.5.1 (QIAGEN, Aarhus, Denmark), following the protocol described by Lasa et al. 2019 [46]. Briefly, reads were quality trimmed based on the quality scores and then length trimmed. Then, reads were clustered at a 97% level of similarity into operational taxonomic units (OTUs), and chimaera detection and removal were performed. Ribosomal RNA gene reads were classified against the nonredundant version of the SILVA SSU reference database (Release 123; http://www.arb-silva.de, accessed on 24 May 2022); [48]). Only reads occurring at least five times in the trimmed dataset were assigned to bacterial taxa and included in the results.

### 4.2. Taxonomic Profiling from RNA-Seq Data

Raw read datasets of a selected number of experiments from our group or data available in public databases were reanalysed and used to perform the following experiments: (1) a comparison to the classic 16S rRNA sequencing methods and (2) a comprehensive comparison of the microbiomes of different *M. galloprovincialis* tissues, immune statuses, and different molluscan species. All the projects used in the current work are listed in Table 1. Prior to the analysis, raw reads (RNA-seq reads) were trimmed to remove sequencing adapters and short and low-quality sequences (quality score limit 0.01 = PHRED 20). Moreover, datasets used for quantitative evaluations were randomly subsampled to equal the lowest number of reads (17 million reads per sample) to compare microbial abundance in a more appropriate manner (each experimental approach is explained in the next sections, and more details on each set of reads are shown in the Supplementary Material).

To analyse the microbiome profiles of the tissues/species referred to before, a working plan consisting of mapping the reads to reference databases was implemented (the whole pipeline is explained in Figure 1). The microorganism reference databases were built with all the virus, protozoan, and fungal genomes deposited in RefSeq up to 18 May 2021. Specifically, these databases contain a total of 10,907 virus assemblies, 94 protozoan assemblies, and 388 fungal assemblies. Additionally, the curated bacterial database included in the CLC Microbial Genomics Module (including 36,027 bacterial assemblies) was used to perform taxonomic profiling. As mentioned above, the software used to perform the taxonomic profiling was the CLC Microbial Genomics Module 21.1 (Qiagen, Hilden, Germany).

Genome assemblies of *Mytilus galloprovincialis* (GCA_900618805.1; [49]), *Pecten maximus* (GCA_902652985.1; [50]), *Haliotis laevigata* (GCA_008038995.1; [51]), and *Octopus vulgaris* (GCA_003957725.1; [52]) were used to filter putative host-specific reads (the approach was the same, and read mapping to each host genome assembly was conducted).

Mapping parameters used to classify read packages in different taxonomic groups were as follows: length fraction = 0.5, similarity fraction = 0.8, and minimum seed length = 30. This minimum seed length parameter defines the minimum perfect match length for a position in the reference to be considered a valid candidate when matching the read.

### 4.3. Differential Abundance Analysis and Alpha Diversity

To determine significant differences in abundance between samples, we performed a differential abundance analysis (the CLC Microbial Genomics Module 21.1 DAA tool). The tool models each abundance table as a separate generalised linear model (GLM), where it is assumed that abundances follow a negative binomial distribution. The Wald test was used to determine significant differences between group pairs and against control group comparisons.

The alpha diversity was calculated using the Vegan R package, allowing us to describe some community ecology parameters. In the current work, Simpson’s index and the Shannon entropy were evaluated in the cases of interest. A pairwise Mann–Whitney U test was also performed to determine which pairs of groups followed different distributions.

### 4.4. Correlation Microbiome/Transcriptome Results

Transcriptomic experiments involving pathogen infections are usually analysed by performing RNA-seq analysis followed by differential expression analysis. However, here, we propose a different approach based on a correlation analysis that may allow us to relate the abundance of a taxon of interest with the expression values obtained by the RNA-seq analysis. In the current work, we performed a Pearson correlation analysis to obtain a collection of genes whose expression values overlapped with the abundance of a concrete taxon.

In the experimental approach considered for this purpose, two infection scenarios with *Vibrio splendidus* took place. In this particular case, the taxon of interest was the Vibrionales order. The abundances of this taxon were compared to the whole transcriptome in both experiments, with retained contigs showing an R^2^ greater than 0.9.

To perform a general comparison of the biological processes modulated/altered by the two different approaches (RNA-seq analysis from Saco et al., 2020 [20], and Rey-Campos et al., 2019 [21], vs. current Pearson correlation), an enrichment analysis was conducted using OmicsBox software (BioBam). Fisher’s exact test was run using a *p* value cut-off of 0.05.

## Figures and Tables

**Table 1:** General information about all the RNA-seq projects used in the current work.

Project	Species	Tissue	Sample Features	Reference
PRJNA858246 PRJNA858248	*Mytilus galloprovincialis*	Whole body	16S rRNA sequencing Ría de Vigo	Current work
PRJNA638821	*Mytilus galloprovincialis*	Gills	Mussel gills after a *V. splendidus* infection by bath	[20]
PRJNA466718	*Mytilus galloprovincialis*	Haemocytes	Mussel haemocytes after a *V. splendidus* injection in the adductor muscle	[21]
PRJNA222492	*Pecten maximus*	Haemocytes	Control from Vigo	[22]
PRJNA481417	*Haliotis diversicolor*	Haemocytes	Control from China	[23]
PRJNA253995	*Octopus vulgaris*	Haemocytes	Control from Vigo	[24]

**Table 2:** Genes correlated with Vibrionales abundance data. Pearson column shows the correlation coefficient.

**Gillbath**			
**ID**	**Pearson**	**Uniprot Annotation**	**Function**
C_8095	−0.993	Regulator of MON1-CCZ1 complex	Endosomal/autophagic flux
C_35153	−0.991	Beta-galactosidase-1-like protein	Probable glycosyl hydrolase
C_53367	−0.991	Zinc finger protein 233	Transcriptional regulation
C_83652	−0.990	tRNA-splicing endonuclease subunit Sen54	Responsible for identification and cleavage of the splice sites in pre-tRNA
C_51191	0.989	AP-5 complex subunit mu-1	May be involved in endosomal transport
C_36214	0.989	NA- and Cl-dependent neutral and basic amino acid transporter B(0+)	Uptake of a broad range of amino acids in a Na+/Cl--dependent manner
C_35181	0.989	Pre-mRNA-splicing factor ISY1 homolog	Processing of microRNAs during embryonic stem cell differentiation
C_55942	−0.987	Protein mab-21-like 3	Neural development
C_63893	−0.986	E3 ubiquitin-protein ligase TRIM56	Role in innate antiviral immunity
C_28519	−0.985	Metabotropic glutamate receptor 3	Signalling via G proteins
C_32331	−0.985	15-hydroxyprostaglandin dehydrogenase	Conversion of hydroxylated arachidonic acid to oxidised metabolites
C_111951	0.985	Retrovirus-related Pol polyprotein from type-2	-
C_94710	−0.985	Chromatin assembly factor 1 subunit A	Chromatin assembly in DNA replication and DNA repair
C_92181	−0.985	Embryonic stem cell-specific 5-hydroxymethylcytosine-binding protein	-
C_17718	−0.984	Ankyrin-1	Attaches integral membrane proteins to cytoskeletal elements
C_21601	−0.984	Sialoadhesin	Acts as an endocytic receptor-mediating clathrin-dependent endocytosis
C_9272	−0.983	Neuronal acetylcholine receptor subunit alpha-10	Ionotropic receptor with a role in the modulation of auditory stimuli
C_36504	−0.983	Innexin unc-9	Plays a role in maintaining gap junction activity to promote locomotion
C_48464	0.983	Neurensin-1	Neural organelle transport
C_22050	−0.983	Cytochrome P450 10	Functions as monooxygenases
**Haemocytes injection**			
**ID**	**Pearson**	**Uniprot Annotation**	**Function**
C_7894	0.997	GTPase IMAP family member 4	Regulation of apoptosis
C_15253	−0.997	Nuclear pore complex protein Nup54	Required for the trafficking across the nuclear membrane
C_13141	−0.997	Bardet–Biedl syndrome 7 protein homolog	Brain development and ciliary trafficking
C_58173	−0.996	Long-chain fatty acid transport protein 3	Long-chain fatty acids translocation at the plasma membrane
C_15154	−0.996	tRNA (uracil-5-)-methyltransferase homolog A	May be involved in nucleic acid metabolism and/or modifications
C_49199	−0.995	Choline dehydrogenase, mitochondrial	Dehydrogenation of choline to betaine aldehyde in mitochondria
C_76285	−0.995	Kelch-like protein 25	Homeostatic mechanism, translational regulation
C_30541	−0.994	4-aminobutyrate aminotransferase, mitochondrial	Catalyses neurotransmitter conversions
C_6950	−0.994	C-Myc-binding protein	Control transcriptional activity of MYC, involved in cell cycle and apoptosis
C_98220	−0.994	UNC93-like protein	Involved in innate and adaptive immune response by regulating TLR signalling
C_52106	−0.991	Programmed cell death protein 2	May play an important role in cell death and/or in regulation of cell proliferation
C_7630	−0.990	GPI inositol-deacylase	Important for efficient transport of GPI-anchored proteins from the ER to the Golgi
C_37669	0.990	Torsin-1A-interacting protein 2	Required for endoplasmic reticulum integrity
C_79722	0.990	Collagen alpha-4(VI) chain	Cell-binding protein
C_18830	−0.990	Copper chaperone for superoxide dismutase	Metalloprotein responsible for the delivery of Cu to SOD1. Antioxidant
C_120023	−0.990	Zinc finger protein 45	May be involved in transcriptional regulation
C_67714	−0.990	Leucine-rich repeat-containing protein 63	Associated with innate immunity. Specially pathogen recognition
C_5776	0.990	Rab9 effector protein with Kelch motifs	Rab9 effector required for endosome to trans-Golgi network (TGN) transport
C_69835	−0.989	TATA box-binding protein-associated factor RNA polymerase I subunit C	Involved in the transcription initiation
C_482	−0.989	Ribonuclease Oy	Releases mononucleotides from RNA in the order of 3’-GMP, 3’-AMP and 3’-UMP

## Data Availability

The datasets generated for this study can be found in the NCBI Short Read Archive database under accession. They are accessible via the BioProject IDs PRJNA858248 (16S-seq A) and PRJNA858246 (16S-seq B).

## Supplementary Materials

The following supporting information can be downloaded at: https://www.mdpi.com/article/10.3390/ijms232416029/s1, Table S1. Methodology comparison. 16S rRNA vs. RNA-seq; Table S2. Sequencing general statistics corresponding to methodology comparison; Table S3. Microbiome comparison between the gills and haemocytes of *Mytilus galloprovincialis*; Table S4. Microbiome changes occurring in the gills and haemocytes of *M. galloprovincialis* after infection; Table S5. Haemocyte microbiomes of different molluscs (*M. galloprovincialis*, *Pecten maximus*, *Haliotis laevigata*, and *Octopus vulgaris*); Figure S1. 16S rRNA sequencing vs. metatranscriptomics using the same samples [19]; Figure S2. Core taxa (order) shared by all gill and haemocyte samples. The numbers above the bars in the histogram indicate the quantity of shared taxa. Red indicates gills, and blue indicates haemocytes; Figure S3. Enrichment analysis comparison between the RNA-seq and correlation approaches; Figure S4. Immune-related genes with expression values that strongly correlated with Vibrionales abundance and were significantly modulated in the RNA-seq analysis.
