# Supplementary material for: Exploring the Potential of Metatranscriptomics to Describe Microbial Communities and Their Effects in Molluscs
Source: Int J Mol Sci. 2022 Dec 16;23(24):16029. doi: 10.3390/ijms232416029 (PMC9784687; doi:10.3390/ijms232416029)
Supplement: Supplementary file 1 [file ijms-23-16029-s001.zip › ijms-2042384-supplementary.pdf]

International Journal of Molecular Sciences

MDPI

Supplementary Material

Table S1. Methodology comparison. 16S rRNA vs RNA-seq reads.

Project	Sequencing	Approach	Tissue	Reference
PRJNA858248<br>PRJNA858246	16S rRNA sequencing	Amplicon-based analysis	Whole body	Current work
PRJNA638821<br>PRJNA466718	RNA sequencing	Taxonomic profiling (RNA-seq reads)	Gills<br>Hemocytes	[20, 21]

Table S2. Sequencing general statistics corresponding to methodology comparison

Sample ID	Sequencing	Raw Reads	Trimmed reads	Mapped Reads
16_25_1 Mussel	16S-seq A	31546	20814	14402
17_15_1 Mussel	16S-seq A	53049	37559	25578
17_25_1 Mussel	16S-seq A	24241	17733	10995
17_35_1 Mussel	16S-seq A	44329	33989	20308
18_15_1 Mussel	16S-seq A	42284	29208	18870
18_25_1 Mussel	16S-seq A	113266	17253	11981
P_23_VB_a	16S-seq B	314326	312099	58769
P_43_VB_a	16S-seq B	303382	302638	51237
P_64_VB_a	16S-seq B	336722	309205	49688
P_88_VB_a	16S-seq B	336340	331256	53183
P_119_VB_a	16S-seq B	355952	313551	44662
P_873_IB_a	16S-seq B	321672	31349	49254
P_902_IB_a	16S-seq B	293076	290484	45314
P_936_IB_a	16S-seq B	325612	282659	41409
P_968_IB_a	16S-seq B	408122	277324	21246
G1	Metatranscriptomics	24096934	24096915	394
G2	Metatranscriptomics	21582336	21582332	206
G3	Metatranscriptomics	19815334	19815330	181
H1	Metatranscriptomics	78426948	78105397	1053
H2	Metatranscriptomics	44346584	43473220	565
H3	Metatranscriptomics	100814198	100400859	3136
H4	Metatranscriptomics	17696894	17357113	3215
H5	Metatranscriptomics	93114098	92741641	1612
H6	Metatranscriptomics	96780602	96432191	2298

Table S3. Microbiome comparison between gills and hemocytes of *Mytilus galloprovincialis*.

Project	Tissue	SRA code	Label at the source job	Current label	Sample features	Reference
PRJNA638821	Gills	SRR11996464	C1	G1	Control mussel	[20]
		SRR11996686	C2	G2	Control mussel	
		SRR11996723	C3	G3	Control mussel	
PRJNA466718	Hemocytes	SRR7138623	C2_t0	H1	Control mussel	[21]
		SRR7138622	C3_t0	H2	Control mussel	
		SRR7138621	C4_t0	H3	Control mussel	
		SRR7138620	I1_t0	H4	Control mussel	
		SRR7138627	I2_t0	H5	Control mussel	
		SRR7138626	I10_t0	H6	Control mussel	

Table S4. Microbiome changes occured in gills and hemocytes of *M. galloprovinciallis* after infection stimuli.

Project	Tissue	SRA code	Label at the source job	Current label	Sample features	Reference
PRJNA638821	Gills	SRR11996464	C1	C1	Control mussel	[20]
		SRR11996686	C2	C2	Control mussel	
		SRR11996723	C3	C3	Control mussel	
		SRR11996734	I1	I1	Infection with *V. splendidus* (waterborne)	
		SRR11996735	I2	I2	Infection with *V. splendidus* (waterborne)	
		SRR11996743	I3	I3	Infection with *V. splendidus* (waterborne)	
PRJNA466718	Hemocytes	SRR7138620	I1_t0	C1	Control mussel	[21]
		SRR7138627	I2_t0	C2	Control mussel	
		SRR7138626	I10_t0	C3	Control mussel	
		SRR7138618	I1_24h	I1	*V. splendidus* injection	
		SRR7138611	I2_24h	I2	*V. splendidus* injection	
		SRR7138610	I10_24h	I3	*V. splendidus* injection	

Table S5. Hemocytes microbiome of different evolutionary-related mollusc (*M. galloprovincialis, Pecten maximus, Haliotis laevigata* and *Octopus vulgaris*).

**Project**	**Species**	**Tissue**	**SRA code**	**Sample features**	**Reference**
PRJNA466718	*M. galloprovincialis*	Hemocytes	SRR7138620-23, SRR7138626-27	Pool of 6 control individuals	[21]
PRJNA222492	*Pecten maximus*	Hemocytes	SRR1009240	Pool of 3 control individuals	[22]
PRJNA481417	*Haliotis diversicolor*	Hemocytes	SRR7529757	Pool of 8 control individuals	[23]
PRJNA253995	*Octopus vulgaris*	Hemocytes	SRR1507221	Pool of 5 control individuals	[24]

Figure S1. 16S rRNA sequencing vs Metatranscriptomics using the same samples [19].

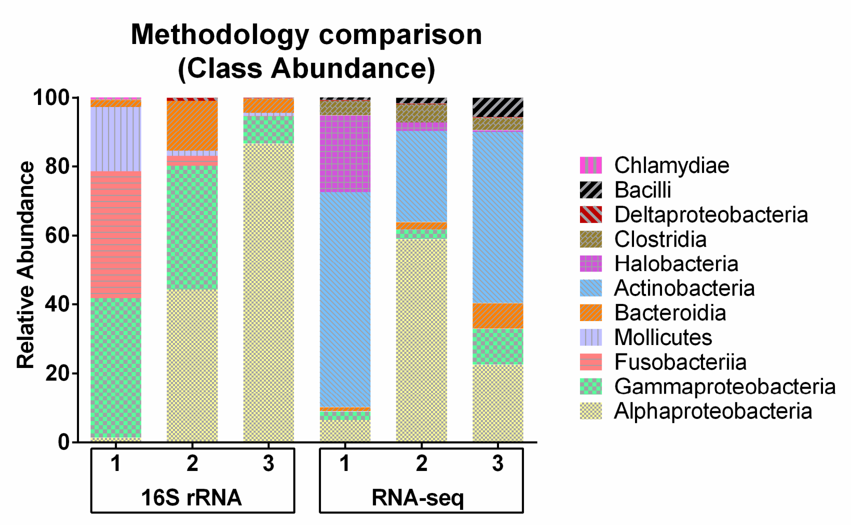

Figure S2. Core taxa (Order) shared by all the gills and haemocytes samples. The numbers above the bars in the histogram indicate the quantity of shared taxa. Red color refers to gills and blue to haemocytes.

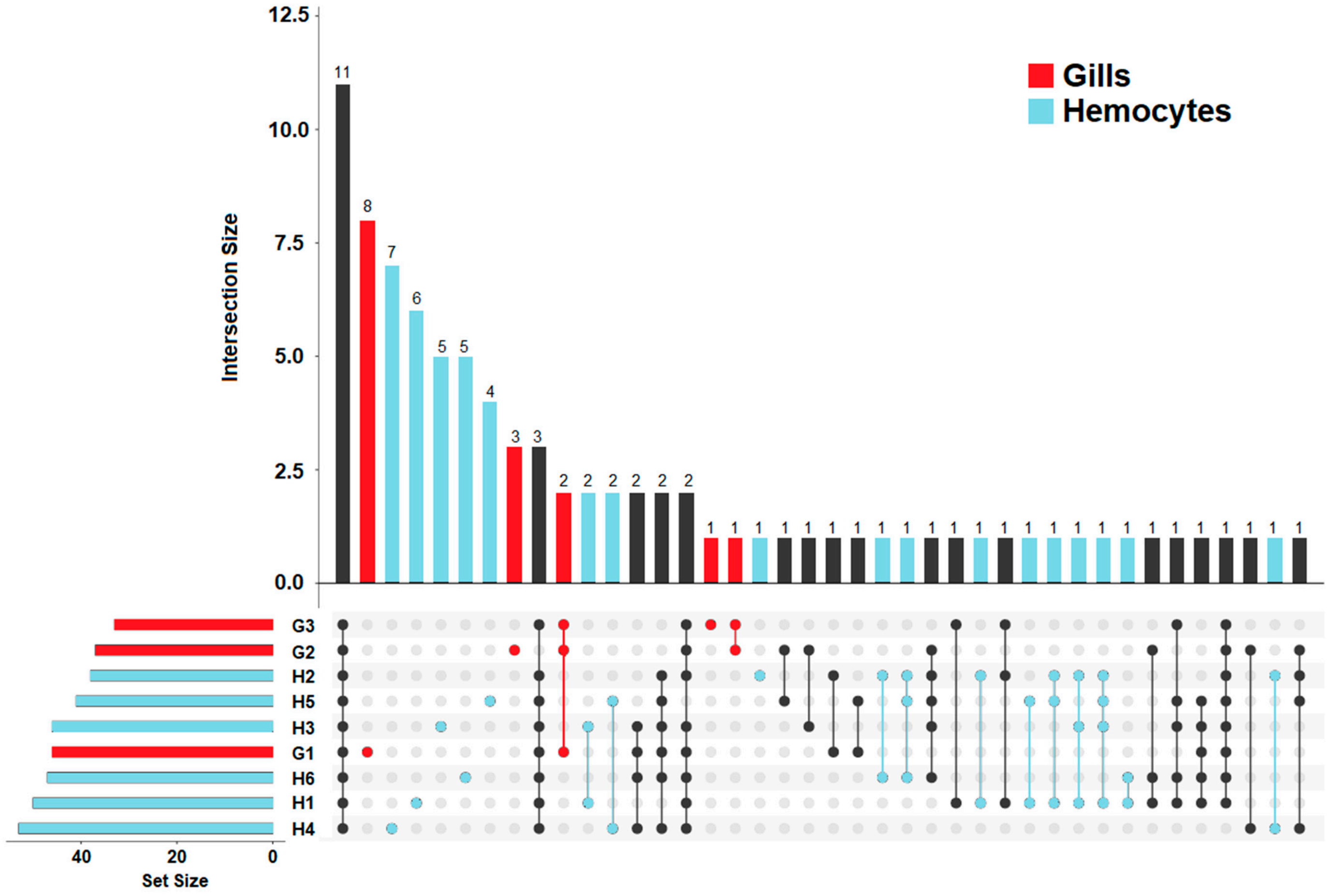

Figure S3. Enrichment analysis comparison between RNA-seq and correlation approaches.

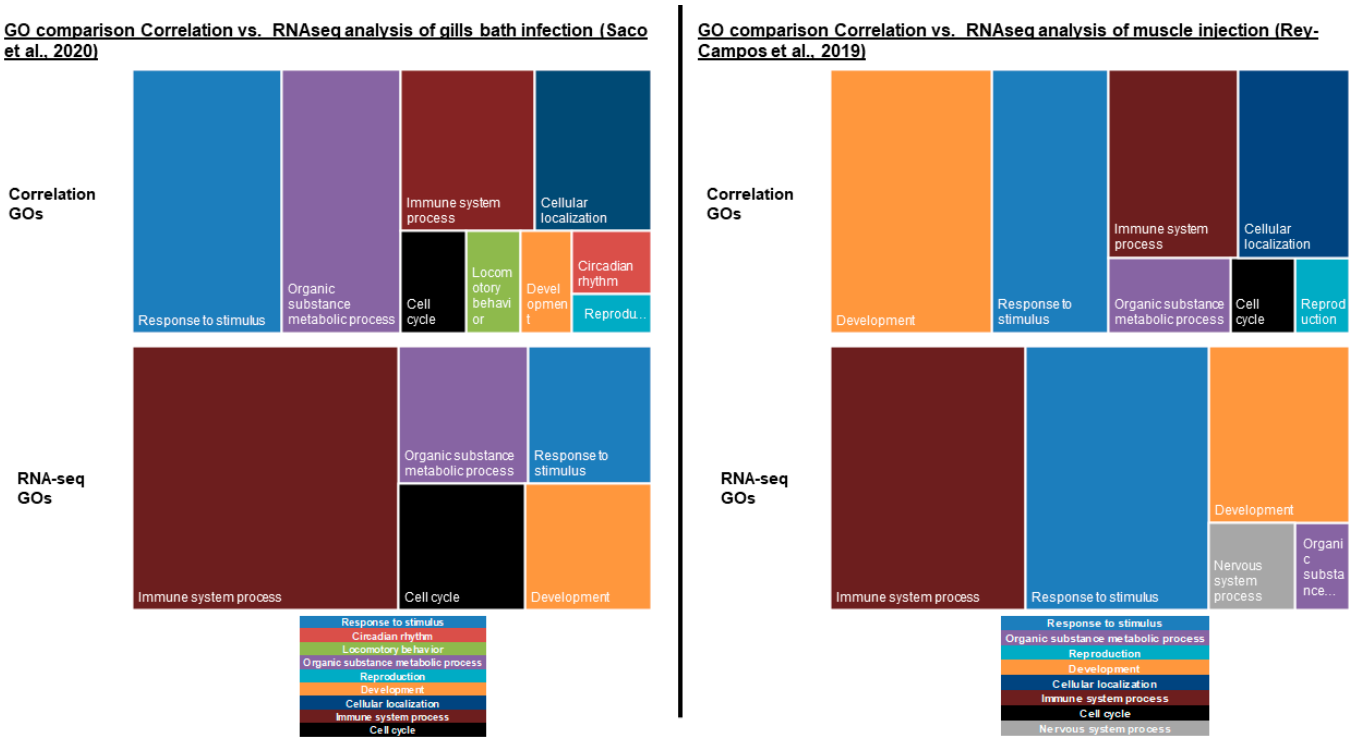

Figure S4. Immune genes showing their expression values strongly correlated to Vibrionales abundance and significantly modulated in the RNA-seq analysis. Table shows immune genes found by correlation.

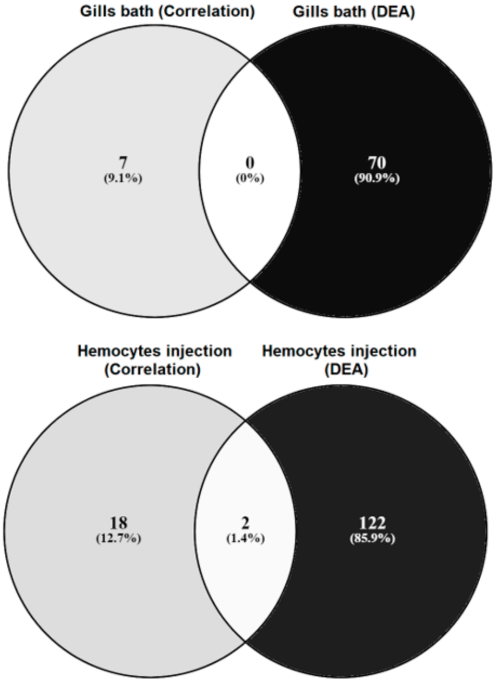

**Gill bath_correlation**	**Uniprot Annotation**
Contig_4201	Toll-like receptor 1
Contig_61747	Pyrokinin-1 receptor
Contig_6057	Transgelin-2
Contig_117165	Protein strawberry notch homolog 1
Contig_9272	Neuronal acetylcholine receptor subunit alpha-10
Contig_21601	Sialoadhesin
Contig_28548	Trans-L-3-hydroxyproline dehydratase
**Hemocytes_correlation**	**Uniprot Annotation**
Contig_6950	C-Myc-binding protein
Contig_8665	B-cell receptor-associated protein 31
Contig_8234	Dynein assembly factor with WDR repeat domains 1
Contig_35752	ATPase family AAA domain-containing protein 1
Contig_7767	Histidine triad nucleotide-binding protein 2, mitochondrial
Contig_3356	E3 ubiquitin-protein ligase MYCBP2
Contig_51931	Histone deacetylase 8
Contig_16754	Transcription intermediary factor 1-beta
Contig_55824	Nibrin
Contig_49344	3-oxo-5-alpha-steroid 4-dehydrogenase 1
Contig_49968	beta-hydroxysteroid epimerase
Contig_23828	Very-long-chain 3-oxoacyl-CoA reductase-B
Contig_106686	Glutathione synthetase
Contig_98411	WD repeat-containing protein 35
Contig_17156	Gamma-glutamyltranspeptidase 1; Short=GGT 1
Contig_24561	Solute carrier family 2, facilitated glucose transporter member 5
Contig_15267	Xanthine dehydrogenase
Contig_69580	Xanthine dehydrogenase/oxidase
Contig_2086	Fos-related antigen 2
Contig_2324	Programmed cell death protein 4
